# The Effects of Amorphous Calcium Carbonate (ACC) Supplementation on Resistance Exercise Performance in Women

**DOI:** 10.3390/nu15030538

**Published:** 2023-01-19

**Authors:** Yitzhak Weinstein, Yarden Ovadia, Bar Weinstein, Ayelet Weinstein

**Affiliations:** 1 Department of Nutritional Sciences, Tel-Hai Academic College, Kiryat Shmone 1220800, Israel; 2 Department of Statistics and Operations Research, Tel Aviv University, Tel Aviv 6997801, Israel; 3 Department of Sports Medicine and Research, Wingate Institute, Netanya 4290200, Israel

**Keywords:** performance, 1-RM, exercise, amorphous calcium carbonate, supplementation

## Abstract

The effects of 9 weeks of amorphous calcium carbonate (ACC) supplementation (1000 mg/day) and resistance exercise training (RT) on one repetition maximum (1-RM) values were tested. Thirty-one women (33.1 ± 7.3 y) were randomly assigned into a supplement (ACC, *n* = 14) or a placebo (PL, *n* = 17) group. On day 1 and following 9 weeks of intervention, the participants underwent anthropometric measurements and filled out a food frequency questionnaire (FFQ) and sports injuries questionnaires. 1-RM values were measured for the back squat and bench press exercises. All the participants significantly (*p* = 0.01) improved their mean back squat and bench press 1-RM values (time effect). While no between-group difference was observed in the bench press 1-RM values, the ACC groups’ mean post-pre bench press 1-RM differences (Δ1-RM) were significantly higher than in the PL group, expressed in kg (*p* = 0.049), per body mass (*p* = 0.042), or per lean body mass (*p* = 0.035). No significant interaction was observed for time X group effect (*p* = 0.421). No differences (within- or between-groups) were observed in the anthropometric values or in the questionnaires’ results. ACC supplementation revealed an ergogenic effect by augmenting the improvement of maximum amount generated force, which can possibly be attributed to the calcium and/or the carbonate components.

## 1. Introduction

Calcium supplementation is often used to attain the recommended calcium intake. However, the effectiveness of calcium supplementation in increasing bone strength is still questionable [1,2,3]. Calcium is a micronutrient that has an important role in both bone health and skeletal muscle function [4]; in addition, it plays an integral role in bone structure and is involved in skeletal muscle function regulation (contraction and relaxation) [5]. A few reviews (i.e., meta-analyses) have concluded that long-term calcium supplementation (over 12 months) is effective in enhancing bone strength; it also has been suggested that this supplementation is more effective when combined with vitamin D [6,7]. Most of the calcium carbonate supplementation studies were carried out on an older population and often combined with vitamin D. Except for Hoffman et al.’s [8] study using amorphous calcium carbonate (ACC) supplementation, there is little research which studies the effectiveness of calcium carbonate supplementation on muscle performance or recovery from exercise in physically active individuals and athletes. 

Calcium supplements are available in either organic or inorganic form, which differentiates their chelating capability [9]. While the organic calcium supplements consist of negatively charged organic molecules (e.g., malate, citrate, and gluconate), the inorganic form includes negatively charged inorganic molecules (e.g., carbonate, phosphates, and chlorides) [9]. Most dietary calcium supplements are made from either carbonate (CO_3_^2−^) or citrate salts that are derived from assorted sources, such as oyster shells, coral calcium, dolomite minerals, and other synthetic material comprised of both organic and inorganic calcium salts [10]. However, the absorbability and bioavailability of calcium from these supplements is limited and is related to the lack of uniformity reported on the efficacy of calcium supplementation [9,11]. Six polymorphs of CaCO_3_ are common minerals in nature. The most stable form of calcium carbonate is calcite (CCC), while the least stable polymorph is the amorphous form (ACC) [12]. Nevertheless, ACC was reported to have a significantly greater absorption capability than CCC [13].

The process of ACC synthesis and transport in freshwater crayfish [14] has led to the development of a novel method for synthetic production of stabilized ACC using phosphor-amino acids [9]. The high bioavailability of ACC over that of CCC has already been demonstrated in both animal [15] and human [16] studies. Furthermore, it was reported that ACC (nano-CaCO_3_) can modulate the acidic environment of cancer tissues and may be used for potential therapeutic purposes, due to its buffering capacity [17].

Various supplements (buffers) have been used by athletes to improve high-intensity performance; among these is sodium bicarbonate (NaHCO_3_), which has been widely used as an effective nutritional anti-acid supplement [18,19]. A meta-analysis reviewed the effects of acute and chronic (5–7 days) NaHCO_3_ ingestion on power generation during high intensity exercise, and concluded that chronic, but not acute, ingestion of NaHCO_3_ increases peak and mean power generation [20]. A major disadvantage of NaHCO_3_ supplementation is the occurrence of gastrointestinal (GI) upset, resulting in symptoms such as nausea, stomach pain, diarrhea, and vomiting [21].

The greater absorption capability and bioavailability of ACC and its potential buffering capacity, as well as the lack of reported GI symptoms associated with its consumption [13] and the limited research on a physically active population [8], provided the rationale for the current research. Thus, the purpose of the current study was to test the effects of 9 weeks of ACC supplementation on one repetition maximum (1-RM) values generated during bench press and back squat exercises in women undergoing resistance training. We hypothesize that ACC supplementation for 9 weeks will lead to a significant improvement in 1-RM values compared with a placebo group.

## 2. Materials and Methods

### 2.1. Experimental Approach

The current study used a randomized, double-blind design. All participants performed the same training protocol, administered by one researcher/instructor (YO), during the entire study. The participants completed two repetition maximum (1-RM) tests during back squat and bench press exercises before and after 9 weeks of ACC/placebo supplementation.

### 2.2. Participants

Thirty-one healthy women were recruited using local social media and participated in the current study, divided randomly (Excel 365 ‘RAND’ and ‘SORT’ functions) into a supplement (ACC; *n* = 14) group or a placebo (PL; *n* = 17) group; their characteristics are detailed in Table 1. Inclusion criteria were defined as: (i) 18–45 years; (ii) do not consume prescription medications; (iii) do not use calcium or hormonal supplementations; (iv) with a regular menstrual cycle. Participants filled out food frequency questionnaires (FFQ) to assess calcium intake (see below), and were instructed not to use any supplements or ergogenic substances during the study period, as well as to adhere to their regular diet. The participants were informed that they were taking part in a study examining resistance exercise performance, and that, as part of the experiment, they would be asked to perform 1-RM tests for back squat and bench press exercises. We excluded participants who were absent from more than 20% of the training sessions; and/or whose supplementation consumption compliance was <85%; and/or who changed their normal diet. Participants with pre-existing illnesses that would impair training and those without a medical approval form were also excluded. The research protocol was approved by the Tel Hai College review board (Tel Hai College IRB 10/2021-8). Informed consent was obtained from all individuals who participated in the current study.

### 2.3. Study Protocol

**Figure 1:**
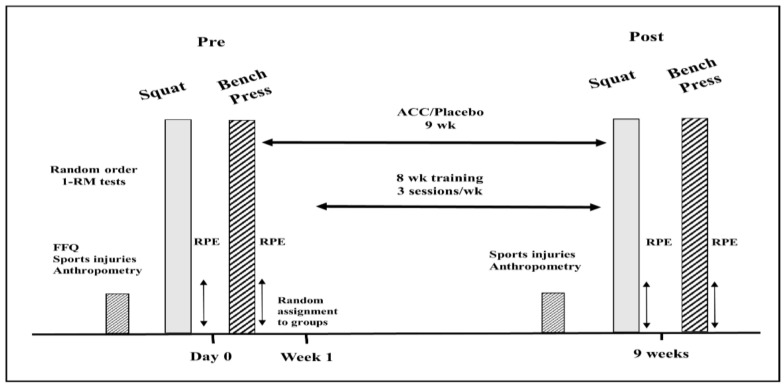
Research protocol and timetable. Pre = beginning of the study; post = following 9-week intervention period; FFQ = food frequency questionnaire; RPE = rate of perceived exertion.

After receiving institutional ethical approval and providing informed consent, all participants signed the consent forms and filled out online physical activity, health history (sport injuries), and FFQ questionnaires. The participants underwent anthropometric measurements, which included weight, height, and body composition analysis, using a Tanita BC-418 Segmental Body Composition Analyzer (Tanita, Tokyo, Japan). Then, the participant performed 1-RM tests for back squat and bench press in a random order. During the 9-week intervention period, all the participants underwent identical training protocols (see below). Following the 9-week intervention/training period, the participants repeated the identical testing procedure that was carried out at the onset of the study (see Figure 1).

### 2.4. Supplement Protocol

ACC and a placebo were supplied for 9 weeks following a double-blind design. The participants received sealed numbered jars containing either ACC or placebo capsules. The participants consumed 1000 mg of slow-release ACC capsules or placebo capsules each day, 5 times/day (200 mg per serving) for 9 weeks, on an empty stomach. There was no difference in the appearance or taste between the active ingredient and placebo capsules.

The ACC capsule consisted of 200 mg ACC (pH Gastrilex^®^), while the placebo (PL) capsule consisted of 200 mg of resistant cellulose. All capsules (ACC and PL) were prepared by Amorphical Ltd., Nes Ziona, Israel. To ensure that the participants consumed the supplements as instructed, they received a weekly supply of capsules (ACC/PL), and their consumption pattern was recorded three times per week during their training sessions. Additionally, the number of weekly capsules consumed was recorded to calculate their compliance to the supplementation during the 9-week intervention period.

### 2.5. Training

After 1 week of the ACC/PL intervention (supplementation), all participants underwent the same training program, consisting of 3 non-adjacent days per week for 8 weeks. The training was administered and supervised by the same researcher (Y.O.), who is a certified fitness instructor. The participants’ initial back squat and bench press resistance during the first week was set at 50% of the individual 1-RM performed at the beginning of the study. Resistance for the other exercises was individually adjusted for each participant by the research personnel (see Table 2).

### 2.6. Maximal Strength Testing

The 1-RM tests were reported suitable for our participants [22] and followed Hoffman et al.’s [23] protocol. All participants completed two 1-RM tests that included back squat and bench press. Each participant performed a warm-up set of 10 repetitions at a resistance equivalent to approximately 50% of the expected 1-RM., Then they performed another warm-up set of five repetitions at a resistance equivalent to approximately 75% of the expected 1-RM. Each participant rested for 3–5 min, followed by performing one repetition with a resistance equivalent to approximately 90–95% of the expected 1-RM. Following another 3–5 min rest, the participant attempted a 1-RM lift and rested for 3–5 min; if the attempt was successful, the resistance was increased, and the participant attempted a new 1-RM lift and continued this protocol until failure. The 1-RM tests (back squat and bench press) were separated by a 30-min rest and administered randomly.

### 2.7. Statistical Analysis

Descriptive statistics were carried out on the participants’ general characteristics, the FFQ and performance questionnaires’ values (1-RM, on BW, and on LBM adjusted 1-RM).

Statistical analysis of performance changes of the supplemented and placebo groups and the times was conducted using a two-way ANOVA (group × time) with repeated measures (RM-ANOVA); the appropriate F-statistics and *p*-values are given in the Section 3. Treatment effects were estimated by contrasting the group’s post-treatment 1-RM values using pre-treatment 1-RM values as covariates. This was carried out by fitting a linear regression model, which followed the formula $Y_{post}=\beta_{0}+\beta_{1}T+\beta_{2}Y_{pre}+\varepsilon$, where $Y_{post},Y_{pre}$ are the post- and pre-intervention 1-RM values, respectively; T is the treatment indicator (***T*** = 1 for ACC group); and ε is a random normal noise. $\beta_{1}$ is the average treatment effect of ACC in comparison to PL, adjusting for pre-period 1-RM values. The analyses’ results were reported as the effect estimate of ACC vs. PL groups in each of the models ($\beta_{1}$). All analyses’ results were reported as the effect estimates, coupled with 95% confidence intervals (CI) and a two-sided *p*-value.

All statistical analyses were performed using the R-Project for Statistical Computing open-source code version 4.1.2 (https://www.r-project.org; accessed on 17 October 2022). The significance level of 5% (*p* < 0.05) was used to determine statistical significance. Data are reported as mean ± SD.

## 3. Results

Anthropometric measurements (mean ± SD) at the beginning of the study are presented in Table 1. No differences were observed between groups or within groups in anthropometric measurements. Mean (±SD) of daily nutritional consumption of micro- and macronutrients obtained from the completed FFQ at the beginning of the study (pre) is shown in Table 3. No significant differences were observed between the ACC and the PL groups in any of the nutritional values.

Mean back squat and bench press exercise 1-RM values produced by both groups at the beginning of the study are shown in Table 4. No differences were observed between the groups in absolute 1-RM values (kg) relative to body mass or relative to lean body mass values (/BW or/LBM, respectively). The results of the RM-ANOVA (Table 5) indicate that within-group (time effect) mean 1-RM values in both exercise modes rose significantly following 8 weeks of training, presented as absolute 1-RM values (kg) relative to body mass or relative to lean body mass values.

**Figure 2:**
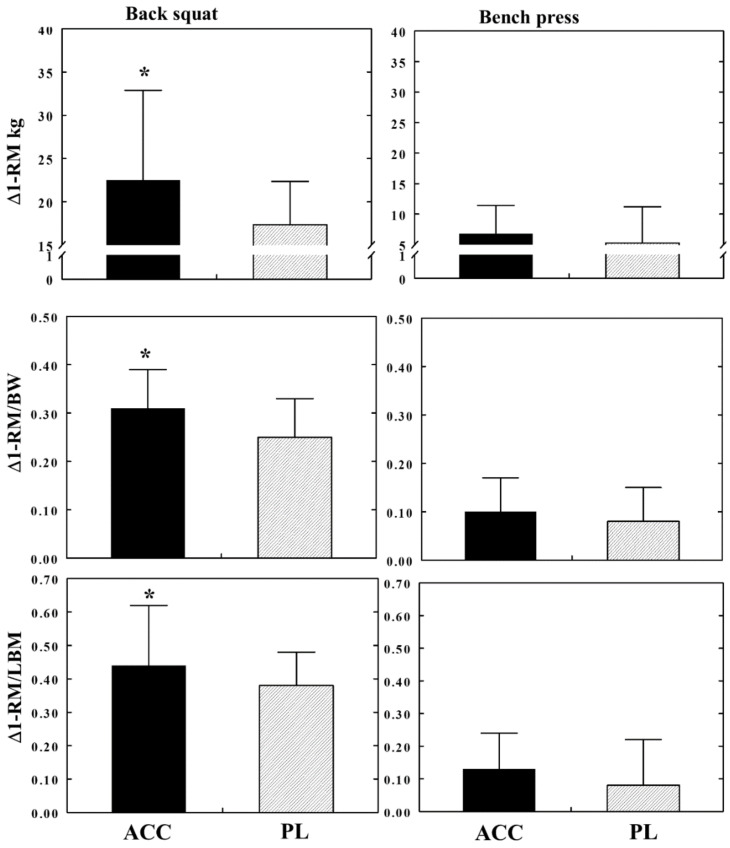
Mean ± SD post-pre 1-RM values (Δ1-RM) for back squat 1-RM exercise (left graphs) and for bench press exercise (right graphs). Values are absolute kg (top graphs; normalized to body weight (BW) (middle graphs) and to lean body mass (LBM) (bottom graphs). * = significant between groups effect (*p* < 0.05). using a linear regression model (see statistical analysis); ACC = ACC group, PL = placebo group.

The RM-ANOVA (Table 5) reveals a significant time effect on absolute back squat 1-RM values, a strong trend for a between-groups effect (*p* < 0.06), and a significant between-groups effects on mass (BW) and LBM-specific back squat 1-RM values. No interactions were found in any of the back squat 1-RM values. No differences were found between the groups in bench press 1-RM values; however, a strong between-group difference trend (*p* < 0.06) was found in absolute bench press 1-RM values. Differences between pre- and post- 1-RM values (Δ1-RM) generated during the back squat and bench press exercises are shown in Figure 2. All back squat Δ1-RM values of the ACC group were significantly higher (*p* < 0.05) than the values exhibited by the PL group.

Table 6 presents 1-RM differences and 95% confidence intervals (CI) showing the effect values (estimated β_1_). Significant effects were found in all back squat Δ1-RM values (absolute, per BW, and per LBM). All effects values for the Δ1-RM performed in the bench press exercise were statistically insignificant.

Analysis of the individual responder/non-responder status in Δ1-RM of the back squat and the bench press exercises indicates that four members of the ACC group exhibited %Δ1-RM greater than 45%, compared with only two participants in the PL group. Four participants in the PL showed >30% in their bench press %Δ1-RM, compared with two participants of the PL group, and the 1-RM bench press value of one participant in the PL group decreased (%Δ1-RM = −13%).

## 4. Discussion

The purpose of the current study was to determine whether a 9-week ACC supplementation can improve 1-RM values generated during bench press and back squat exercises in women undergoing resistance training. The main finding of the study was a significant 1-RM improvement in the back squat exercise in the ACC group compared with the PL group, whether expressed in absolute values (kg), normalized to body mass (BW), or to lean body mass (LBM) to minimize the large variance of BW, LBM, and 1-RM values. The lack of a significant difference between groups in 1-RM values generated in the bench press exercise following the intervention period may be explained by the smaller muscle mass of those participants performing this task [22]. Janssen et al. [24] reported that 58% of the muscle mass of women is in the lower body; thus, the performance difference by the upper body muscles was smaller and insignificant.

To the best of our knowledge, there are no published studies that determine the ergogenic effect of ACC supplementation. Therefore, we compared our results with studies using sodium bicarbonate (NaHCO_3_) supplementation [18,19]. A meta-analysis reviewed the effects of acute and chronic (5–7 days) NaHCO_3_ ingestion on power generation during high-intensity exercise and concluded that chronic (5–7 days), but not acute, ingestion of NaHCO_3_ increases peak and mean power generation [20]. In the current study, the supplementation period was extended to 9 weeks to overlap the training duration, as was recommended by β-Ala supplementation effects on a performance position statement [25]. Santana et al. [26] reported improvement in 10-km running time following 23 days of β-Ala supplementation, and Van Thienen et al. [27] showed an 11.4% improvement in mean power output following 8 weeks of β-Ala supplementation.

The overall improvement in the 1-RM values among all participants (within group) in the current study is a result of the well-controlled training. Considering that most of the participants began the study with relatively low muscle strength (i.e., mean back squat 1-RM values = 53.8 ± 11.5 kg), this was an expected outcome [28]. The augmented performance exhibited by the ACC group compared with the PL group may be attributed to the mechanisms of calcium ion and/or to the carbonate ion of the ACC molecule. The greater absorption capability and bioavailability of ACC (Ca^2+^) [15] and its potential buffering capacity via the HCO_3_^−^ system, as well as the strong ion difference (SID) [29], may offer a partial explanation to the mechanism of the supplement, which brought about the improved performance by the ACC group.

Calcium supplementations has generally not been considered ergogenic [4]. Traditional calcium supplementation reversed the risk for fractures among athletes who performed high-volume exercise, which causes a decline in calcium levels [30]. Nevertheless, the superior absorbability and stability of ACC [9,15,16] may increase the possibility of an ergogenic effect. While it is known that most of the body’s calcium is stored in the bones [31], its role in muscle performance is not well understood. Increased calcium availability may lead to rising mitochondrial calcium uptake [32]. The importance of mitochondrial calcium’s role in the control of metabolism and ATP synthesis and numerous physiological functions has been reviewed by Mammucari et al. [32]. It has been reported that extracellular Ca^2+^ interacts with lowered trans-sarcolemmal K^+^ gradients during muscle contractions, where a reduction in extracellular Ca^2+^ lowers the intracellular activity of K^+^ and contributes to the development of muscle fatigue [33]. Furthermore, increased calcium (a strong ion) levels may contribute to improved acid-base status regulation by increasing the value of the strong ion difference (SID), thus acting to reduce acidity [29,34]. The exact mechanism which could explain the positive effect of the calcium component of the ACC supplementation on back squat 1-RM performance in the current study is unclear.

Considering the role of the carbonate component of the ACC, Hoffman et al. [8] concluded that ACC supplementation may have potentially lessened the decline in performance, which is possibly due to the carbonate component of the ACC. Since there are no published studies on the ergogenic effects of ACC, we again examined studies which investigated NaHCO_3_ supplementation effects on performance. It has been reported that NaHCO_3_ has an ergogenic effect by buffering the rise in hydrogen ions levels produced during high-intensity exercise in lower body, but not in upper body, exercise [35]. Similar to the results of our study, it has been shown that a greater number of repetitions to failure were achieved in the back squat, but not in the bench press, exercise following an acute ingestion of NaHCO_3_ [36]. However, it should be noted that the contribution of the sodium ion (Na^+^), which is a very strong base in aqueous solutions [29], to the acid-base status was not discussed in these or in other NaHCO_3_ supplementation studies.

Bicarbonate comprises ∼95% of the body’s total CO_2_ content (TCO_2_) [37]. TCO_2_ is a dependent acid-base variable, and its value is determined by the value of CO_2_ partial pressure (PCO_2_) in the body’s liquids [29]. The addition of CO_3_^2−^ to the body’s fluids will most likely lead to a minor increase in PCO_2_. However, the increased TCO_2_ can enhance the buffering capacity of the bicarbonate system, which may partially explain the improved performance. The exact mechanism which explains the positive effect of the CO_3_^2−^ component of the ACC supplementation on performance in the current study is also unclear and awaits further investigation.

The current study is the first to examine possible ergogenic effects of ACC supplementation. Since all the participants underwent an identical training program, administered and supervised by the same research team personnel, close supervision of the participants’ adherence to ACC/placebo consumption was possible. Study limitations include the fact that measuring initial calcium blood levels before and after the study period might have been valuable in better understanding daily calcium intake and response to the supplementation.

## 5. Conclusions

In conclusion, long-term ACC supplementation may have an ergogenic effect on maximal force generation. Future studies should focus on ACC supplementation effects in various sports events and investigate the mechanisms of ACC action, which may explain its ergogenic property.

## Figures and Tables

**Table 1:** Participants’ characteristics for the ACC (*n* = 14) and the PL (*n* = 17) groups. *p* values are generated from a two-sided independent samples *t*-test.

Variable	ACC	PL	*p*-Value
Age (years)	32.3 ± 7.7	33.6 ± 7.2	0.765
BM (kg)	67.1 ± 16.0	72.6 ± 18.1	0.302
Height (cm)	162.5 ± 4.9	165.5 ± 5.4	0.131
BMI (kg/m^2^)	25.4 ± 6.4	26.4 ± 6.0	0.662
LBMI (kg/m^2^)	16.5 ± 2.2	16.5 ± 1.5	0.972
BF (%)	31.9 ± 8.1	35.6 ± 9.1	0.258
LBM (kg)	43.5 ± 4.6	45.3 ± 4.9	0.306

ACC = amorphous calcium carbonate; PL = placebo; BM = body mass; BMI = body mass index; LBMI = lean body mass index; BF = body fat percentage; LBM = lean body mass (e.g., FFM).

**Table 2:** Eight-week resistance training program.

Exercise	Week 1–2 (Sets × Reps)	Week 3–4 (Sets × Reps)	Week 5–6 (Sets × Reps)	Week 7–8 (Sets × Reps)
Barbell back squat	4 × 8–10	4 × 10–12	5 × 10–12	5 × 12–14
Rubber band pull-ups	4 × 10–12	4 × 12–15	4 × 12–15	5 × 12–15
Bench press	4 × 8–10	4 × 10–12	5 × 10–12	5 × 12–14
Push press	-	4 × 8–10	4 × 10–12	4 × 12–15
Standing shoulder press	4 × 8–10	4 × 8–10	4 × 10–12	4 × 10–12
Static squat battle rope shake	4 × 40–60	4 × 40–60	4 × 60–70	4 × 60–70
Biceps curls	4 × 8–10	4 × 10–12	5 × 10–12	5 × 12–14
Romanian deadlift	-	4 × 8–10	4 × 10–12	5 × 10–12
TRX narrow row	4 × 8–10	4 × 8–10	4 × 10–12	4 × 10–12
Saw dumbbell single hand row	4 × 10–12	4 × 12–15	4 × 12–15	5 × 12–15
Wall ball throw	4 × 10–12	4 × 12–15	4 × 12–15	4 × 12–15
Abdominal routine	3 × 10	3 × 12	4 × 10	4 × 12

Reps = number of repetitions in each set.

**Table 3:** Mean (±SD) daily micro- and macronutritional consumption values of the ACC and the PL groups, derived from the FFQ completed at the beginning of the study (two-tailed *t*-test).

Group	ACC	PL	
Variable, Units/Day	(*n* = 14)	(*n* = 17)	*p*-Value
Energy, Kcal	2723.3 ± 1159.4	2914.5 ± 1018.3	0.943
Protein, g	114.0 ± 54.0	132.1 ± 54.8	0.738
Fat, g	113.8 ± 50.2	104.9 ± 36.5	0.585
CHO, g	285.4 ± 146.9	303.9 ± 132.2	0.725
Water, g	3754.5 ± 1390.9	4409.5 ± 1280.5	0.198
Calcium, mg	1281.5 ± 602.0	1535.2 ± 732.4	0.551
Vitamin D, µg	8.9 ± 6.4	11.3 ± 7.2	0.354

CHO = carbohydrates.

**Table 4:** Mean ±SD baseline 1-RM values performed by the ACC (*n* = 14) and the PL (*n* = 17) groups (two-tailed *t*-test).

Exercise	ACC	PL	*p*-Value
Squat, kg	55.5 ± 12.4	52.4 ± 11.3	0.471
Squat, kg/kg BW	0.88 ± 0.3	0.76 ± 0.2	0.213
Squat, kg/kg LBM	1.24 ± 0.3	1.12 ± 0.2	0.199
Bench Press, kg	39.3 ± 10.3	38.2 ± 5.6	0.749
Bench Press, kg/kg BW	0.59 ± 0.1	0.54 ± 0.1	0.259
Bench Press, kg/kg LBM	0.87 ± 0.2	0.83 ± 0.2	0.602

BW = body weight (kg); LBM = lean body mass (kg); C

**Table 5:** RM-ANOVA analysis of 1-RM performance analysis by the ACC (*n* = 14) and the PL (*n* = 17) groups before and following (pre-post) 8 weeks of training and nutritional intervention. Degrees of Freedom for all analyses = 1, 58.

Exercise	Group	*p*-Value	Time	*p*-Value	Gr X T	*p*-Value
Squat abs	3.53	0.065	44.06	0.001 *	0.66	0.421
Squat/BW	5.61	0.021 *	19.14	0.001 *	0.22	0.638
Squat/LBM	7.85	0.007 *	40.58	0.001 *	0.77	0.385
Bench Press abs	0.64	0.429	7.48	0.008 *	0.11	0.738
Bench Press/BW	3.63	0.062	10.05	0.002 *	0.16	0.691
Bench Press/LBM	1.88	0.176	5.30	0.025 *	0.31	0.580

Group = F values for group effect; Time = F values for time effect; Gr X T = F values for group X Time (interaction) effect; BW = body weight (kg) and LBM = lean body mass (kg); * *p* < 0.05.

**Table 6:** Mean treatment effect and 95% confidence intervals (CI) between the ACC (*n* = 14) and the PL (*n* = 17) groups.

Exercise	Effect	95% CI	*p*-Value
Squat	5.62	[0.27, 10.96]	0.049 *
Squat/BW	0.07	[0.01, 013]	0.042 *
Squat/LBM	0.14	[0.02, 0.26]	0.035 *
Bench Press	1.57	[−2.29, 5.43]	0.432
Bench Press/BW	0.03	[−0.03, 0.08]	0.337
Bench Press/LBM	0.06	[−0.02, 0.15]	0.128

BW = body weight (kg); LBM = lean body mass (kg); * *p* < 0.05.

## Data Availability

The data presented in this study are available on request from the corresponding author.

## References

[1] Bischoff-Ferrari H.A., Dawson-Hughes B., Baron J.A., Burckhardt P., Li R., Spiegelman D., Willett W.C. (2007). Calcium intake and hip fracture risk in men and women: A meta-analysis of prospective cohort studies and randomized controlled trials. Am. J. Clin. Nutr..

[2] Bolland M.J., Leung W., Tai V., Bastin S., Gamble G.D., Grey A., Reid I.R. (2015). Calcium intake and risk of fracture: Systematic review. BMJ.

[3] Tang B.M., Eslick G.D., Nowson C., Smith C., Bensoussan A. (2007). Use of calcium or calcium in combination with vitamin D supplementation to prevent fractures and bone loss in people aged 50 years and older: A meta-analysis. Lancet.

[4] Arroyo E., Jajtner A.R., Hoffman J.R. (2019). Vitamins and Minerals. Dietary Supplementation in Sport and Exercise.

[5] Harvey N.C., Biver E., Kaufman J.M., Bauer J., Branco J., Brandi M.L., Cooper C. (2017). The role of calcium supplementation in healthy musculoskeletal ageing: An expert consensus meeting of the European Society for Clinical and Economic Aspects of Osteoporosis, Osteoarthritis and Musculoskeletal Diseases (ESCEO) and the International Foundation for Osteoporosis (IOF). Osteoporos. Int..

[6] Chandran M., Tay D., Mithal A. (2019). Supplemental calcium intake in the aging individual: Implications on skeletal and cardiovascular health. Aging Clin. Exp. Res..

[7] Weaver C.M., Alexander D.D., Boushey C.J., Dawson-Hughes B., Lappe J.M., LeBoff M.S., Looker A.C., Wallace T.C., Wang D.D. (2016). Calcium plus vitamin D supplementation and risk of fractures: An updated meta-analysis from the National Osteoporosis Foundation. Osteoporos. Int..

[8] Hoffman J.R., Ben-Zeev T., Zamir A., Levi C., Ostfeld I. (2022). Examination of amorphous calcium carbonate on the inflammatory and muscle damage response in experienced resistance trained individuals. Nutrients.

[9] Meiron O.E., Bar-David E., Aflalo E.D., Shechter A., Stepensky D., Berman A., Sagi A. (2011). Solubility and bioavailability of stabilized amorphous calcium carbonate. J. Bone Miner. Res..

[10] Straub D.A. (2007). Calcium supplementation in clinical practice: A review of forms, doses, and indications. Nutr. Clin. Pract..

[11] Heaney R.P., Dowell M.S., Bierman J., Hale C.A., Bendich A. (2001). Absorbability and cost effectiveness in calcium supplementation. J. Am. Coll. Nutr..

[12] Nebel H., Neumann M., Mayer C., Epple M. (2008). On the structure of amorphous calcium carbonate--a detailed study by solidstate NMR spectroscopy. Inorg. Chem..

[13] Gal J.Y., Bollinger J.C., Tolosa H., Gache N. (1996). Calcium carbonate solubility: A reappraisal of scale formation and inhibition. Talanta.

[14] Shechter A., Berman A., Singer A., Freiman A., Grinstein M., Erez J., Sagi A. (2008). Reciprocal changes in calcification of the gastrolith and cuticle during the molt cycle of the red claw crayfish *Cherax quadricarinatus*. Biol. Bull..

[15] Shaltiel G., Bar-David E., Meiron O.E., Waltman E., Shechter A., Aflalo E.D., Sagi A. (2013). Bone loss prevention in ovariectomized rats using stable amorphous calcium carbonate. Health.

[16] Vaisman N., Shaltiel G., Daniely M., Meiron O.E., Shechter A., Abrams S.A., Sagi A. (2014). Increased calcium absorption from synthetic stable amorphous calcium carbonate. J. Bone Mineral Res..

[17] Som A., Raliya R., Tian L., Akers W., Ippolito J.E., Singamaneni S., Achilefu S. (2016). Monodispersed calcium carbonate nanoparticles modulate local pH and inhibit tumor growth in vivo. Nanoscale.

[18] De Oliveira L.F., Saunders B., Artioli G.G. (2018). Is bypassing the stomach a means to optimize sodium bicarbonate supplementation? A case study with a postbariatric surgery individual. Int. J. Sport Nutr. Exerc. Metab..

[19] Hadzic M., Eckstein M.L., Schugardt M. (2019). The impact of sodium bicarbonate on performance in response to exercise duration in athletes: A systematic review. J. Sports Sci. Med..

[20] Lopes-Silva J.P., Reale R., Franchini E. (2019). Acute and chronic effect of sodium bicarbonate ingestion on Wingate test performance: A systematic review and meta-analysis. J. Sports Sci..

[21] Burke L.M., Pyne D.B. (2007). Bicarbonate loading to enhance training and competitive performance. Int J. Sports Physiol. Performance..

[22] Seo D.I., Kim E., Fahs C.A., Rossow L., Young K., Ferguson S.L., So W.Y. (2012). Reliability of the one-repetition maximum test based on muscle group and gender. J. Sports Sci. Med..

[23] Hoffman J.R. (2014). Physiological Aspects of Sport Training and Performance.

[24] Janssen I., Heymsfield S.B., Baumgartner R.N., Ross R. (2000). Estimation of skeletal muscle mass by bioelectrical impedance analysis. J. Appl. Physiol..

[25] Trexler E.T., Smith-Ryan A.E., Stout J.R., Hoffman J.R., Wilborn C.D., Sale C., Antonio J. (2015). International society of sports nutrition position stand: Beta-Alanine. J. Int. Soc. Sports Nutr..

[26] Santana J.O., De Freitas M.C., Dos Santos D.M., Rossi F.E., Lira F.S., Rosa-Neto J.C., Caperuto E.C. (2018). Beta-alanine supplementation improved 10-km running time trial in physically active adults. Front. Physiol..

[27] Van Thienen R., Van Proeyen K., Puype J., Lefere T., Hespel P. (2009). Beta-alanine improves sprint performance in endurance cycling. Med. Sci. Sports Exerc..

[28] Lopez P., Radaelli R., Taaffe D.R., Newton R.U., Galvão D.A., Trajano G.S., Teodoro J.L., Kraemer W.J., Häkkinen K., Pinto R.S. (2021). Resistance training load effects on muscle hypertrophy and strength gain: Systematic review and network meta-analysis. Med. Sci. Sports Exerc..

[29] Stewart P.A. (1983). Modern quantitative acid–base chemistry. Can. J. Physiol. Pharma..

[30] Lappe J., Cullen D., Haynatzki G., Recker R., Ahlf R., Thompson K. (2008). Calcium and vitamin d supplementation decreases incidence of stress fractures in female navy recruits. J. Bone Miner. Res..

[31] Peacock M. (2010). Calcium metabolism in health and disease. Clin. J. Am. Soc. Nephrol..

[32] Mammucari C., Raffaello A., VecellioReane D., Gherardi G., De Mario A., Rizzuto R. (2018). Mitochondrial calcium uptake in organ physiology: From molecular mechanism to animal models. Pflügers Archiv Europ. J. Physiol..

[33] Cairns S.P., Leader J.P., Loiselle D.S., Higgins A., Lin W., Renaud J.M. (2015). Extracellular Ca^2+^-induced force restoration in K^+^-depressed skeletal muscle of the mouse involves an elevation of [K^+^] i: Implications for fatigue. J. Appl. Physiol..

[34] Lindinger M.I. (2021). Total carbon dioxide in adult standardbred and thoroughbred Horses. J. Equine Veter. Sci..

[35] McNaughton L.R., Gough L., Deb S., Bentley D., Sparks S.A. (2016). Recent developments in the use of sodium Bicarbonate as an ergogenic aid. Curr. Sports Med. Rep..

[36] Duncan M.J., Weldon A., Price M.J. (2014). The effect of sodium bicarbonate ingestion on back squat and bench press exercise to failure. J. Strength Cond. Res..

[37] Burton R.F. (1987). On calculating concentrations of ”HCO_3_“ from pH and PCO_2_. Comp. Biochem. Physiol. A Comp. Physiol..

